# Whole-genome sequencing of African swine fever virus from wild boars in the Kaliningrad region reveals unique and distinguishing genomic mutations

**DOI:** 10.3389/fvets.2022.1019808

**Published:** 2023-01-05

**Authors:** Ali Mazloum, Antoinette van Schalkwyk, Andrey Shotin, Nikolay Zinyakov, Alexey Igolkin, Roman Chernishev, Zoran Debeljak, Fedor Korennoy, Alexander V. Sprygin

**Affiliations:** ^1^Reference Laboratory for African Swine Fever Virus, FGBI “Federal Centre for Animal Health” (FGBI “ARRIAH”), Vladimir, Russia; ^2^Agricultural Research Council - Onderstepoort Veterinary Institute, Pretoria, South Africa; ^3^Department of Biotechnology, University of the Western Cape, Bellville, South Africa; ^4^Department of Epizoology, Veterinary Specialist Institute “Kraljevo”, Kraljevo, Serbia

**Keywords:** African swine fever, molecular evolution, phylogenetic analysis, genome markers, whole-genome sequence

## Abstract

**Introduction:**

Since the first report of outbreaks of African swine fever (ASF) in Georgia in 2007, the disease has expanded into Europe, Russia, and Asia, spreading rapidly *via* contact with infected animals including domestic pigs and wild boars. The vast expansion of this Genotype II African swine fever virus (ASFV) across wide-ranging territories and hosts inevitably led to the acquisition of novel mutations. These mutations could be used to track the molecular epidemiology of ASFV, provided that they are unique to strains restricted within a certain area. Whilst whole-genome sequencing remains the gold standard for examining evolutionary changes, sequencing of a single locus with significant variation and resolution power could be used as a rapid and cost-effective alternative to characterize multiple isolates from a single or related outbreak.

**Material and methods:**

ASFVs obtained during active ASF outbreaks in the Russian region of Kaliningrad between 2017 and 2019 were examined. Since all of the viruses belonged to Genotype II and no clear differentiation based on central variable region (CVR) sequencing was observed, the whole-genome sequences of nine ASFV isolates from this region were determined. To obtain insights into the molecular evolution of these isolates, their sequences were compared to isolates from Europe, Asia, and Africa.

**Results:**

Phylogenetic analysis based on the whole-genome sequences clustered the new isolates as a sister lineage to isolates from Poland and Germany. This suggests a possible shared origin followed by the addition of novel mutations restricted to isolates from this region. This status as a sister lineage was mirrored when analyzing polymorphisms in MGF-505-5R and MGF-110-7L, whilst a polymorphism unique to sequences from Kaliningrad was identified at locus K145R. This newly identified mutation was able to distinguish the isolates obtained from Kaliningrad with sequences of Genotype II ASFVs available on GenBank.

**Discussion:**

The findings of this study suggest that ASFVs circulating in Kaliningrad have recently obtained this mutation providing an additional marker to the mutations previously described.

## Introduction

African swine fever (ASF) is a contagious viral disease of pigs and wild boars, with a fatality rate reaching 100% in susceptible animals. It typically manifests as a hemorrhagic fever, but can present in various forms from subclinical to hyperacute (1). No effective preventive measures nor treatments against ASF have yet been established (1). The infectious agent of ASF is an eponymous arbovirus of the A*sfarviridae* family, called African swine fever virus (ASFV). It has a large double-stranded DNA genome ranging from 170 to 190 kilobase pairs (kbp) that encodes more than 150 open reading frames (ORFs), depending on the viral strain (2). Owing to a sylvatic cycle involving natural hosts including warthogs and ticks, ASFV is currently the only known DNA arbovirus (3, 4).

Historically, ASF was first identified in the early 1900s in East Africa, but rapidly received recognition as a transboundary disease with significant economic impact (5). The first expansion of infection beyond the African continent occurred in 1957 and 1960 when ASFV genotype I spread to Spain and Portugal, along with sporadic outbreaks in Western Europe, Latin America, and the Caribbean. The disease became endemic in Sardinia, but was eradicated from the Iberian Peninsula by the 1990s (6). The second major transcontinental spread occurred in 2007, when outbreaks caused by ASFV genotype II were reported in Georgia (7, 8). Following this incursion, ASFVs belonging to genotype II spread extensively throughout Europe, Russia, Asia, and the Greater Antilles archipelago (9). In 2021, the first outbreak of ASF caused by a virus belonging to genotype I was reported in China, but these viruses shared high identity with the genotype I strains from Spain and Portugal in the 1960s (10).

Asymptomatic reservoirs of wild animals and insect vectors play an important role in the epidemiology of ASF (11). The established reservoirs of the virus in Africa include warthogs, bush pigs, and ticks of the genus *Ornithodoros* (12), whereas in northern latitudes, wild boars constitute the main source of persistence. The natural range of wild boars extends from Western Europe and the Mediterranean basin to eastern Russia, Japan, and Southeast Asia (13). ASF is not considered endemic to wild boars in Russia (14); however, considering their intensive reproduction rate, hidden way of life (15), nocturnal activity (16), and migration over longer distances across shared land borders, these free-ranging ASFV carriers are now attracting increased research interest as an important player in ASF epidemiology and molecular evolution. Considering these aspects, this study focused on phylogenetic and evolutionary analyses of ASFVs obtained from wild boars.

Phylogenetic studies into ASFV evolution are critical for establishing relationships among circulating isolates and tracking origins of outbreaks. The variable C-terminal region of the *B646L* gene is an appropriate target to resolve outbreaks and assign ASFVs to one of the 24 known genotypes (17). Similarly, the central variable region (CVR) located in gene B602L and SNPs in E183L were used to resolve ASFVs obtained from outbreaks in Africa and Europe (18, 19). Unfortunately, analyses based on B646L, B602L, and E183L genes are insufficient to differentiate genotype II isolates currently circulating in a single country or specific region in Eurasia (20). Based on phylogenomic analysis of the genotype II viruses circulating in Europe, Russia, and Asia since 2007, the inability of single locus sequences to differentiate between ASFVs appears to stem from a genetic bottleneck due to the founder effect imposed on the ASFV population (21). Subsequent mutations are associated with genetic drift and/or duplication and/or deletion events of repeat regions (21–23). Initial studies based on sequence analysis using only B646L, E183L, I196L, B602L, and KP86R gene regions, as well as the intragenic regions (IGRs) between I73R/I329R and I78R/I215L, suggested that a single ASFV variant caused the outbreaks from 2007 to 2011 in Russia (21). In contrast, phylogenetic analysis using whole-genome sequences of ASFVs isolated from Russia between 2014 and 2019 indicated clear genetic divergence between isolates obtained in the west and east of the country (24). The eastern isolates were phylogenetically related to samples obtained in Poland, China, Belgium, and Moldova, while the western isolates had single-nucleotide polymorphisms (SNPs) identical to those in the original Georgia/2007, Lithuania, and genotype II isolates from Africa. This phylogenetic separation between eastern and western isolates was based on the whole-genome sequences as well as informative SNPs described in genes MGF-360-10L, MGF-505-9R, and I267L as well as within the IGR between I173R and I329R (24). Whole-genome sequencing is therefore imperative for identifying novel mutations, which can subsequently be used to investigate the evolution and epidemiology of ASFVs. In this regard, to obtain a complete understanding of the molecular evolution of ASFV in Russia, it is necessary to analyze isolates from all possible administrative units and search for other resolution markers.

The Kaliningrad region of Russia is the only Oblast that shares no borders with other Russian regions and is located between ASF-affected Lithuania and Poland. In this region, ASF outbreaks were regularly reported between 2017 and January 2020 (9), but in the absence of whole-genome sequence analyses of the isolates, an in-depth examination of the ASFV molecular epidemiology in Europe is required.

In this study, we report the results of whole-genome sequencing of nine ASFV isolates from wild boars in the Kaliningrad region from 2017 to 2019, as well as in-depth comparison to sequences from Europe and Asia.

## Materials and methods

### Study area

**Figure 1:**
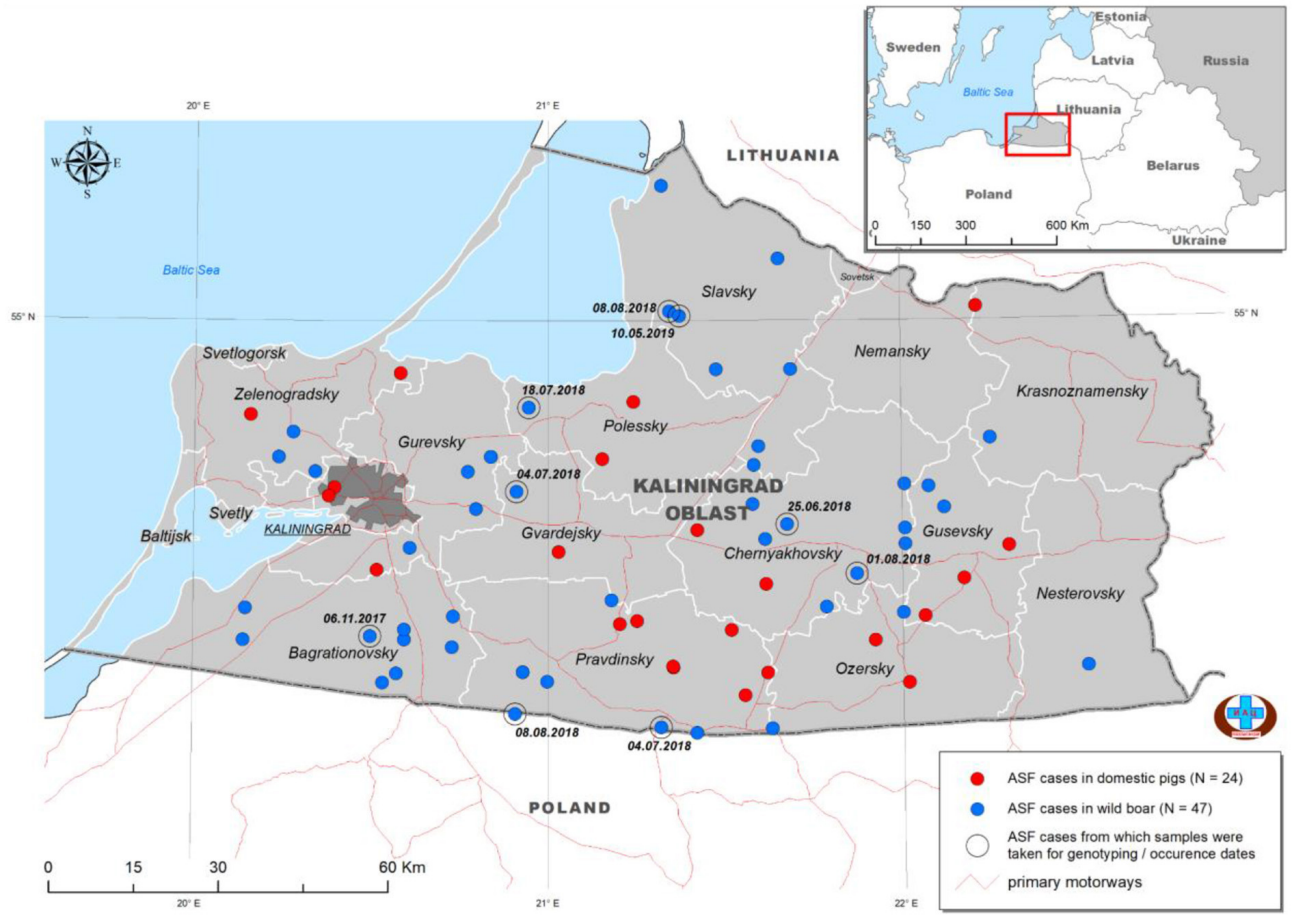
Distribution of ASFV outbreaks and cases in Kaliningrad between 2017 and 2019. The ASF-positive outbreaks from domestic pigs (*n* = 24) are indicated by red dots, whilst those from wild boars (*n* = 47) are indicated by blue dots. The nine isolates used in this study are highlighted by a second circle and include the date of notification (data from official OIE site as of 25/3/2022).

Kaliningrad is a 223 km^2^ Oblast of the Russian Federation (RF) between Lithuania and Poland on the Baltic Sea (Figure 1). The first outbreaks of ASF in Kaliningrad were reported in 2017. Between 2017 and 2019, 71 samples from the Kaliningrad region were submitted to the OIE reference center at FGBI-ARRIAH for laboratory confirmation of ASF outbreaks. Of the 71 ASFV PCR-positive samples, nine were selected based on their spatial and temporal distribution within the Kaliningrad region for whole-genome sequencing (Table 1; Figure 1). Figure 1 presents a map of all reported outbreaks within this region during 2017–2019. This study focuses on nine ASF clinical samples from deceased wild boars that were subsequently selected for analysis based on unique spatial and temporal distributions. The average distance between the locations of the outbreaks corresponding to the nine selected samples was 25 km (Figure 1).

**Table 1:** Brief characteristics of the ASFV strains used in this study.

**Sample name**	**Tissue**	**Date**	**Location**	**Coordinates of outbreak site**
ASFV/Kaliningrad_17/WB-13869	Bones from carcass	07-11-2017	Bagrationovsky near the village of Krasnoarmeiskoye	54.48, 20.50
ASFV/Kaliningrad_18/WB-9766	Spleen	08-07-2018	Polessky district, Ovrazhye settlement	54.86, 20.94
ASFV/Kaliningrad_18/WB-12523	Spleen	07-08-2018	Slavsky district, Zapovednoe	55.01, 21.37
ASFV/Kaliningrad_18/WB-9735	Spleen	03-07-2018	Pravdinsky district	54.34, 21.32
ASFV/Kaliningrad_18/WB-9734	Spleen	25-06-2018	Chernyakhovskoye district, Kamensk	54.66, 21.67
ASFV/Kaliningrad_18/WB-9763	Bones from carcass	07-07-2018	Guards district, near the village of Osinovka, Osinovskoe	54.72, 20.91
ASFV/Kaliningrad_18/WB-12524	Spleen	30-07-2018	Pravdinsky district, Shirokoye	54.36, 20.91
ASFV/Kaliningrad_18/WB-12516	Spleen	07-08-2018	Chernyakhovskoye district, near the village of Shuvalovo	54.58, 21.87
ASFV/Kaliningrad_19/WB-10168	Spleen	13-05-2019	Slavsky district, near the village of Zapovedni	55.01, 21.37

### Samples

Samples from domestic pigs (*n* = 24) and deceased wild boars (*n* = 47) were submitted to the OIE Reference Laboratory for ASF [Federal Center for Animal Health, (FGBI-ARRIAH), Vladimir, Russia] for laboratory confirmation of ASF. The samples were homogenized and diluted in sterile PBS to a 10% suspension for use in downstream applications. Total DNA was extracted using a DNA mini kit, in accordance with the manufacturer's instructions (Qiagen, Germany). The presence of ASFV DNA was detected using real-time PCR in accordance with the recommendations of the OIE (25). The metadata of the nine samples selected for this study are provided in Table 1.

### Virus isolation

Nine samples of the PCR-positive submissions from wild boar (*n* = 47) were selected for further virus isolation (VI) (Table 1). One hundred microliters of the previously prepared 10% homogenate tissue samples were used for VI, in accordance with the previously described protocol (24). In brief, the viruses were isolated using primary porcine spleen cell culture (PSC) in 10 mL cell suspension [Eagle's minimal essential medium (MEM) containing 10% fetal bovine serum (FBS), 0.008% gentamicin, 10^6^ U penicillin, and 0.1 g/mL streptomycin] in a T25 cell culture flask at 37°C for 7 days. The propagated strains were passaged three times on PSC (Supplementary Table 1) before harvesting and genomic viral DNA extraction.

### Whole-genome sequencing

Total genomic DNA (gDNA) of the ASFV isolates was extracted using the phenol-chloroform method as described by Szpara et al. (26) with some modifications. Briefly, each virus was grown on PSC cell culture in six T-25 flasks for 3 days, when the cells were collected by centrifugation at 3,000 g for 30 min at 4°C, following confirmation by hemadsorption. Each pellet was resuspended in cold, filtered PBS (Sigma) and frozen at −70°C, prior to repeated freeze/thaw cycles at −70 and 37°C in order to lyse the cells. The cellular debris was removed by centrifugation at 3,000 g for 10 min at 4°C, and the supernatant was moved to a new tube. The supernatant was treated with 0.25 U/μL DNase I (Evrogen) and 20 μg/mL RNAse A (Evrogen) and incubated for 1 h at 37°C, followed by the addition of protease from *Streptomyces griseus* (Sigma) to a final concentration of 10 U/ml and incubation at 37°C overnight. The mixture was centrifuged using a 30% sucrose cushion at 15,890×g for 30 min at 4°C and the pellet was resuspended in 300 μL of PBS. Viral capsid was lysed by adding NaCl (final concentration of 1 M), 200 μg/mL proteinase K (Sigma), and SDS (final concentration 1%) and incubated for 1 h at 56°C, followed by two extractions using phenol-chloroform. After centrifugation at 7,000 g for 3 min, the upper layer was collected, to 200 μL of which we added 10 μL of 3 M NaAc (pH 5.5) and 2 volumes of ice-cold 100% ethanol. The mixture was gently inverted and incubated at −70°C for 3 h, followed by centrifugation at 12,000 g for 30 min at 4°C. The gDNA pellet was washed with 70% ethanol and resuspended in 50 μl of TE buffer. The quality and quantity were measured using a spectrophotometer (Eppendorf). A sequencing library was constructed for each of the samples using the Nextera XT DNA library preparation kit (Illumina, USA) and next-generation sequencing (NGS) was performed using a MiSeq reagent kit version 2 with 2 × 250-bp paired-end sequencing on a MiSeq benchtop sequencer (Illumina, USA). Between 650,000 and 7,255,000 reads were obtained for each of the samples. The quality of the reads was determined and adaptors as well as low-quality reads were removed using CLC Genomics Workbench v9 (Qiagen, www.clcbio.com). To assemble the genome, high-quality trimmed reads were mapped to the reference genome (FR682468.2_ASFV/Georgia 2007/1) using CLC Genomics Workbench v9. New consensus sequences were obtained for each of the samples from mapping to the reference sequence. Additionally, the high-quality trimmed reads were *de novo* assembled and the resulting consensus sequence for each sample was compared to its corresponding consensus sequences obtained from the mapping analysis in CLC Genomics Workbench v9. No discrepancies were observed between the two individual consensus sequences of each sample and the original consensus sequences were used in subsequent analysis. The trimmed reads were further mapped to their corresponding new consensus virus genomes with an average coverage depth >50× using CLC Genomics Workbench v9 (Table 2).

**Table 2:** Whole-genome sequencing data for all nine ASFV isolates from Kaliningrad and GenBank accession numbers.

**Sample name**	**Genome size**	**Total number of reads**	**Percentage of reads mapped to ASFV consensus sequence (%)**	**Average coverage**	**GenBank accession number**
ASFV/Kaliningrad_17/WB-13869	189,315	7,255,000	73	>100	OM799941
ASFV/Kaliningrad_18/WB-9766	189,344	933,597	84	>100	OM966718
ASFV/Kaliningrad_18/WB-12523	189,373	4,575,000	65	>100	OM966714
ASFV/Kaliningrad_18/WB-9735	189,392	650,000	58	60–120	OM966716
ASFV/Kaliningrad_18/WB-9734	189,221	710,000	53	70–130	OM966721
ASFV/Kaliningrad_18/WB-9763	189,304	2,874,000	41	>100	OM966717
ASFV/Kaliningrad_18/WB-12524	189,374	2,120,000	82	>100	OM966715
ASFV/Kaliningrad_18/WB-12516	189,194	765,000	88	51–110	OM966720
ASFV/Kaliningrad_19/WB-10168	189,389	3,888,000	75	>100	OM966719

Open reading frames (ORFs) were predicted using ASFV/Georgia 2007/1 (FR682468.2) as the reference in the GATU software and the whole-genome sequences were submitted to GenBank (Table 2).

### Phylogenetic and single-nucleotide polymorphism (SNP) analysis

To perform genetic analysis and comparison of isolates from the RF and neighboring countries, data of previously sequenced ASFV isolates were obtained from GenBank (Supplementary Table 2). These included sequences from Estonia in 2014, China from 2018 to 2020, Poland from 2015 to 2019, Belgium in 2018, Czech Republic in 2017, Moldova in 2017, Lithuania in 2014, Vietnam in 2019, Germany in 2020, Ukraine in 2016, Hungary in 2018, and the revised sequence of the 2007 case in Georgia, ASFV/Georgia 2007/1 (FR682468.2), as well as five published isolates from different regions of the RF (Supplementary Table 2). Additionally, genotype II sequences from Africa (Tanzania in 2017 and Malawi in 2019) as well as the 2019 outbreak in Timor-Leste were included in all of the analyses (Supplementary Table 2). These sequences were used to generate an alignment, detect single-nucleotide polymorphisms (SNPs), and determine the phylogenetic relatedness of the isolates (Table 2). The alignment construction and SNP detection were performed using CLC Genomics Workbench v.9 based on the statistical alignment algorithms describe by Hein (27).

Confirmation of SNP's in ORF K145R was performed by Sanger sequencing as described previously by Mazur-Panasiuk et al. (28).

The best fit model was predicted for each of the phylogenetic analyses in Mega X. Phylogenetic analysis of the sequences was performed by generating a maximum likelihood tree, with 1000 bootstrap iterations under the General Time Reversible (GTR, *G* + I = 4) model in Mega X.

## Results

### Whole-genome analysis

Nine PCR-positive ASFV samples from the Kaliningrad region of the RF were selected, based on their spatial and temporal distributions, for whole-genome sequencing (Table 1; Figure 1). These isolates were propagated in PSCs where they showed hemadsorption and an increase in virus titer which confirmed virus replication (Supplementary Table 1). Later, infected cell culture was used for viral genomic DNA extraction and Nextera XT library construction for sequencing on the MiSeq platform (Illumina). Between 650,000 and 7.255 million reads were obtained and consensus sequences for each isolate were generated with a calculated average coverage depth >50× for all nine isolates. The genome length of sequenced isolates varied between 189,194 and 189,392 bp (Table 2).

**Figure 2:**
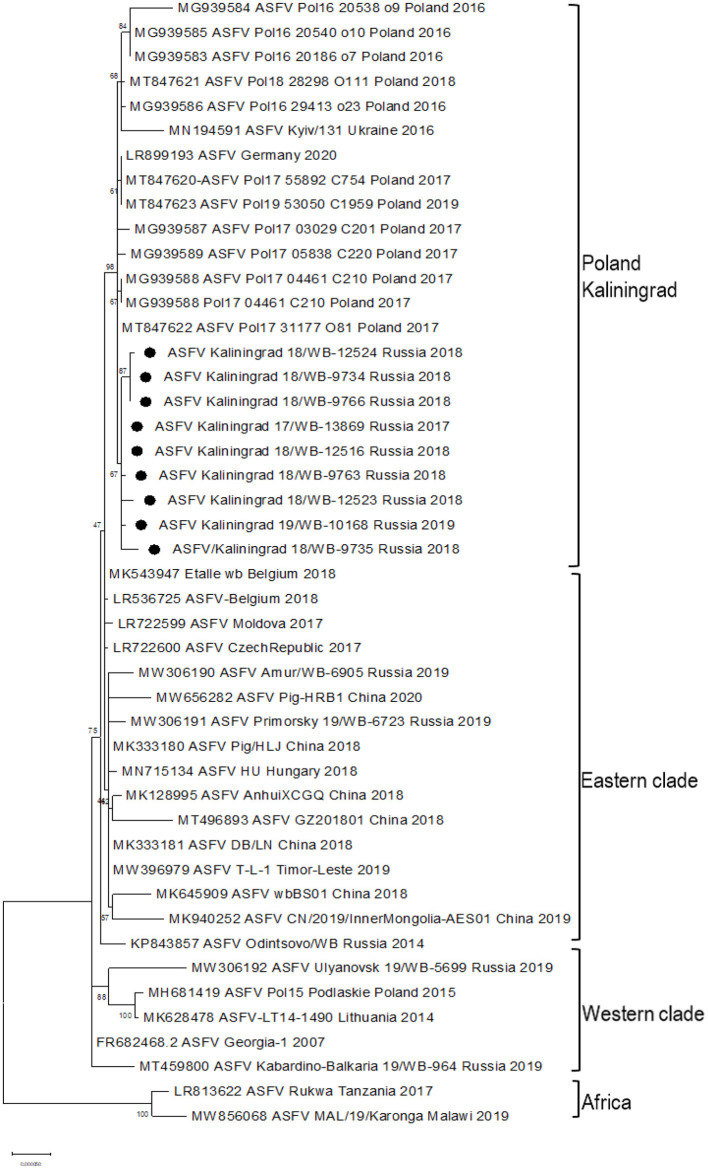
Maximum likelihood phylogenetic tree of the whole-genome sequences of selected ASFV genotype II. Sequences from isolates obtained in Kaliningrad are indicated with black circles. The sequences from Kaliningrad, Poland, Germany, and the Ukraine cluster are in a unique sister lineage to the previously described eastern and western clades.

Based on the ~413 bp C-terminal region of ORF B646L, comparisons were made between ASFV sequences representing genotype II from Africa, Europe, and Asia (Supplementary Table 2) as well as 21 of the remaining 23 genotypes. As expected, all nine samples clustered with genotype II based on the B646L (p72) gene region (data not shown). Further sub-genotyping analysis was performed based on the CVR (B602L) of the nine isolates showing a 100% identity with reference isolate Georgia 2007/1. An alignment consisting of the whole-genome sequences from genotype II isolates in Africa, Europe, and Asia (Supplementary Table 2) as well as the nine new sequences from Kaliningrad was generated and used either as the whole genome or according to selected gene regions in subsequent phylogenetic analysis. Based on the whole-genome sequences, phylogenetic analysis indicated that the genotype II isolates from Europe and Asia clustered into three possible groups (Figure 2). The first group consisted of isolates from Germany/2020, Ukraine/2016, the nine samples from Kaliningrad, and 12 isolates from Poland between 2016 and 2018 (Figure 2). The other two groups were previously described to contain samples representing the western or eastern region of Russia (24).

The whole-genome sequences of the new isolates from Kaliningrad were compared against the ASFV genome sequences listed in Supplementary Table 2 and various SNPs were identified (Table 3). Ten novel nonsynonymous and four synonymous SNPs were described between the isolates of Kaliningrad and the other ASFVs (Table 3). The majority of these SNPs affected one or two of the nine isolates, whilst two synonymous and two nonsynonymous ones were identified in all nine of the Kaliningrad sequences (Table 3). These are located in MGF-505-5R (I330V), MGF-110-7L (S20S), and two in K145R (Y145S and Y194Y) (Table 3). Based on the four SNPs identified in ORFs MGF-505-5R, MGF-110-7L, and K145R, they would be included in the subsequent phylogenetic analysis based on a single locus.

**Table 3:** Amino acid positions affected by the synonymous (=) and nonsynonymous (<) SNPs in the predicted proteins.

**Protein **	**Position: Amino acid **	**ASFV/Kaliningrad_19/WB-10168 **	**ASFV/Kaliningrad_18/WB-9766 **	**ASFV/Kaliningrad_18/WB-12523 **	**ASFV/Kaliningrad_18/WB-9735 **	**ASFV/Kaliningrad_17WB-13869 **	**ASFV/Kaliningrad_18/WB-9763 **	**ASFV/Kaliningrad_18/WB-12524 **	**ASFV/Kaliningrad_18/WB-12516 **	**ASFV/Kaliningrad_18/WB-9734 **	**MT847620_ASFV_Pol55892_C754_Poland_2017 **	**Georgia/2007-1** ** (FR682468.2) **
MGF-360-1L	147: D < E	E	E	E	E	**D**	E	E	E	E	E	E
ASFV-G-ACD-0090	5: F < S	S	S	**F**	S	S	S	S	S	S	S	S
MGF 110-13L	117: G < W	W	W	W	W	W	**G**	W	W	W	W	W
MGF 505-5R	330: I < V	I	I	I	I	I	I	I	I	I	I	V
MGF 505-5R	414: R < K	K	K	K	K	K	K	**R**	K	K	K	K
K145R	97: Y = Y	T	T	T	T	T	T	T	T	T	C	C
K145R	145: Y < S	Y	Y	Y	Y	Y	Y	Y	Y	Y	Y	S
EP402R	87: N < H	H	H	H	**N**	H	H	H	H	H	H	H
EP402R	140: N < Y	Y	Y	Y	**N**	Y	Y	Y	Y	Y	Y	Y
G1211R	904: K < E	E	**K**	E	E	E	E	**K**	E	E	E	E
E199L	127: R < G	**R**	G	G	G	G	G	**R**	G	G	G	G
MGF 110-7L	20: S = S	A	A	A	A	A	A	A	A	A	A	G
MGF 505-6R	134: C = C	C	C	**T**	C	C	C	C	C	C	C	C
K205R	166: L = L	T	T	**C**	T	T	T	T	T	T	T	T
K145R	97: Y = Y	T	T	T	T	T	T	T	T	T	C	C

The SNPs were identified between the nine isolates from Kaliningrad, Georgia/2007-1 (FR682468.2), and ASFV/Pol55892/C754/Poland/2017 (MT847620). The amino acid position as well as the composition of each sequence at a position are indicated with the polymorphism in bold. The four predicted proteins in gray contain informative SNPs capable of differentiating the isolates from Kaliningrad from the original Georgia/2007-1 (FR682468.2).

### Single-gene analysis

Whole-genome sequence alignments were used to investigate individual single gene loci, selected based on either previous studies or new SNPs identified within this study.

### MGF-360-10L, MGF-505-9R, and I267L

Since neither B646L nor the CVR located within the B602L gene (results not shown) was capable of fine-scale discrimination within genotype II, additional gene regions were investigated in the subsequent phylogenetic analysis. The ORFs MGF-360-10L, MGF-505-9R, and I267L as well as the intragenic regions (IGR) between I733R and I329R and between MGF-505-9R and MGF505-10R were examined, based on a previously reported study (24). Previous analysis divided the isolates obtained from the east and west of Russia into eastern and western clades by using the ORFs MGF-360-10L, MGF-505-9R, and I267L (24). Inclusion of the new sequences from Kaliningrad and Poland grouped these within the western clade using MGF-360-10L, but the eastern clade using MGF-505-9R and I267L (Supplementary Figures 1A–C).

### The IGR between I173R and I329L

**Figure 3:**
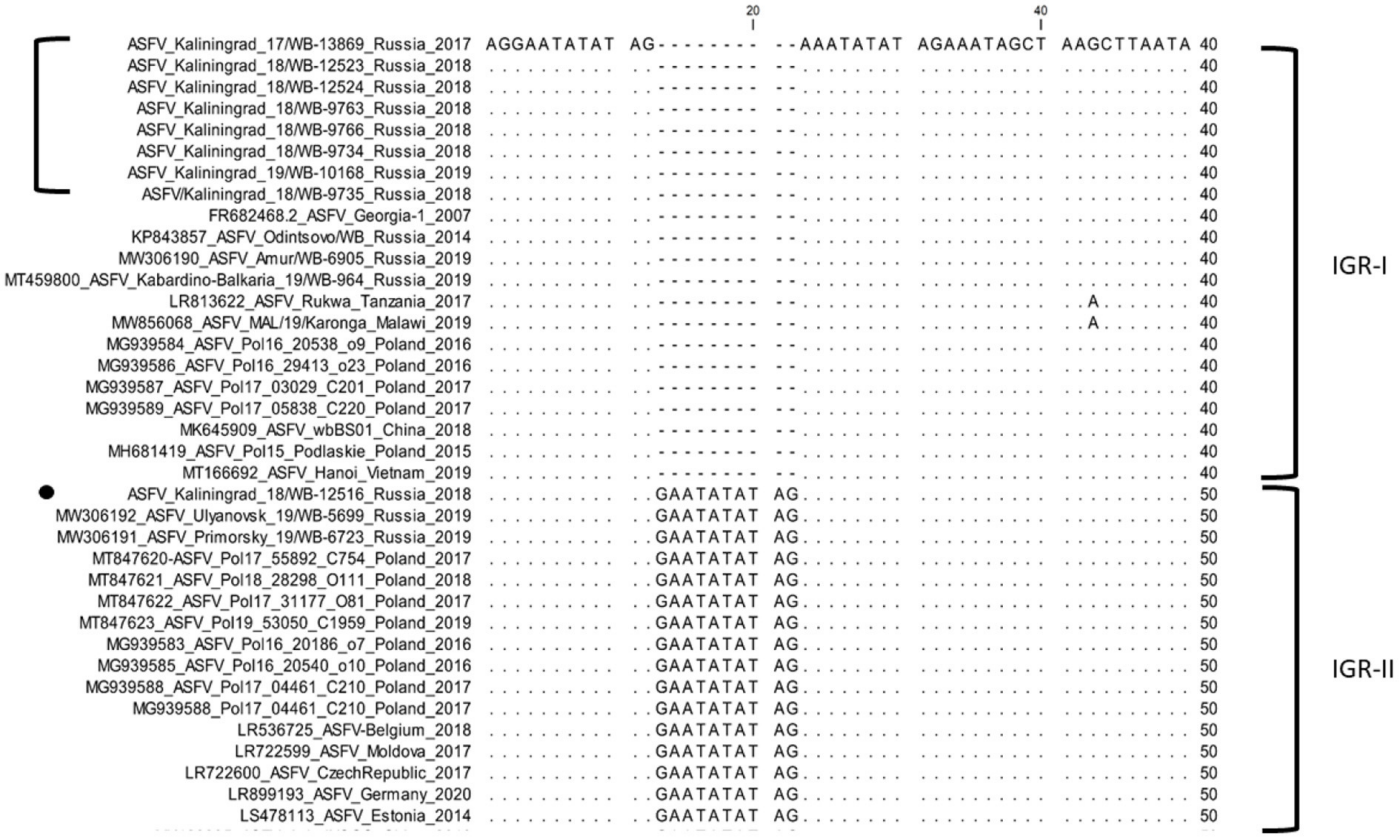
Nucleotide sequence alignment of the intergenic region between I73R and I329L genes of ASFV strains belonging to either cluster IGR-I or cluster IGR-II.

In contrast, the IGR between I173R and I329L grouped isolates from Amur, Kabardino-Balkaria, and eight isolates from Kaliningrad into cluster I (IGR-I), whilst isolates from Primorsky and Ulyanovsk were clustered into cluster (IGR-II) along with a single isolate from Kaliningrad “ASFV/Kaliningrad_18/WB-12516” (Figure 3).

### MGF-505-5R and MGF-110-7L

**Figure 4:**
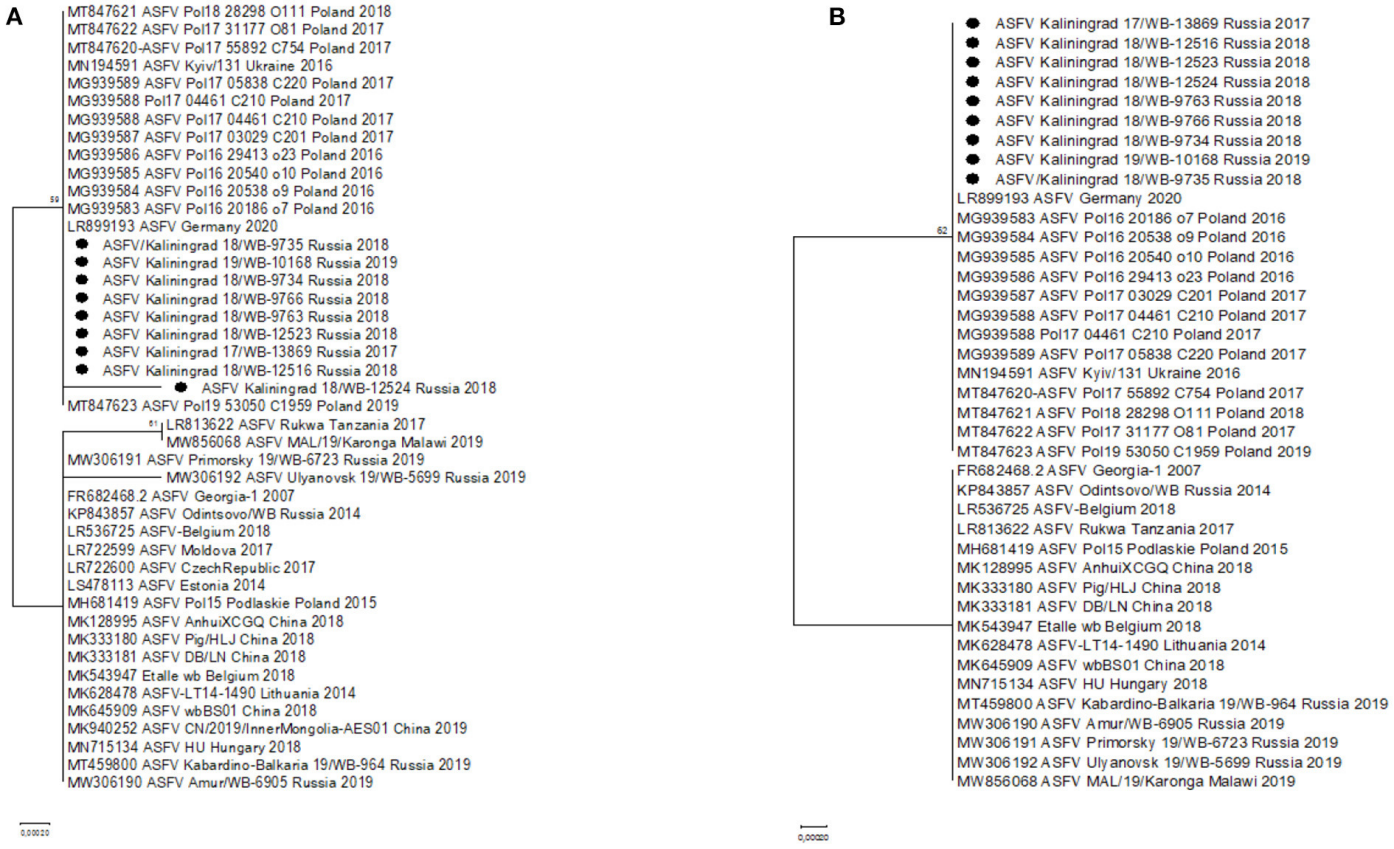
Maximum likelihood phylogenetic tree indicating the relationship of ASFV genotype II sequences, using selected ORFs, MGF-505-5R **(A)** and MGF-110-7L **(B)**. The nine isolates from Kaliningrad are indicated with black circles.

Two SNPs identified in ORFs MGF-505-5R (I330V) and MGF-110-7L (S20S) were unique to the sequences from Kaliningrad, in comparison to the previously analyzed sequences from the RF (Table 3). This led to these ORFs being subsequently selected as possible markers for additional discrimination across the ASFV genotype II strains. The ORFs were used in phylogenetic analysis and, based on MGF-505-5R and MGF-110-7L, sequences from Kaliningrad clustered with sequences from Poland and Germany in 2020 and the Ukraine in 2016 in a unique cluster with the remainder of the genotype II ASFVs (Figure 4).

### ORF K145R

**Figure 5:**
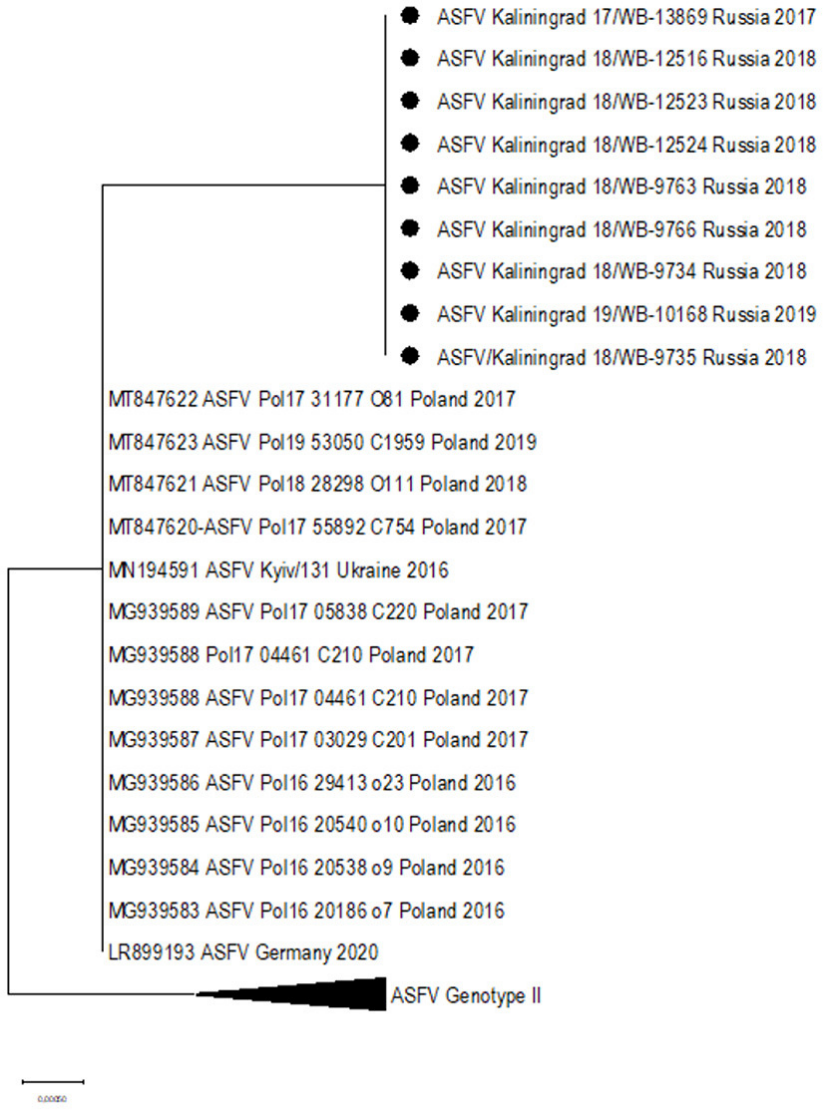
Maximum likelihood phylogenetic tree indicating the relationship of ASFV isolates based on ORF K145R.

Additionally, two SNPs unique to the sequences from Kaliningrad in comparison to the previously analyzed sequences from the RF were identified in K145R, C > T at position 291 and C > A at position 434 (Table 4). Phylogenetic analysis based on K145R grouped the isolates from Kaliningrad into an unique cluster, while sequences from Poland grouped into a sister cluster with the sequences from Germany and the Ukraine, and finally the rest of the ASFV genotype II sequences grouped into a third cluster (Figure 5). The two SNP's detected in this ORF were further confirmed by Sanger sequencing as described previously (Results not shown) (28). This implies that ORF K145R could possibly be used to distinguish samples from Kaliningrad from those from the rest of Europe and Asia.

**Table 4:** Clustering of individual isolates based on SNPs or indels observed in the IGR between I73R and I329L, as well as the ORFs O174L, K145R, and MGF-505-5R.

**No**.	**Isolate**	IGR/I73R -I329L^*^	O174L^**^	K145R^***^	MGF 505-5R^****^	Group^*****^

		**TRS (10 nt.)**	**TRS (14 nt)**	**291: C** > **T** **434: C** > **A**	**988: G** > **A**	
1	FR682468.2 ASFV Georgia 2007/1	I	I	I	I	1
2	ASFV/Kabardino-Balkaria 19/WB-964	I	I	I	I	1
3	ASFVOdincovo2019	I	I	I	I	1
4	ASFV/Amur 19/WB-6905	I	I	I	I	1
5	China ASFV-wbBS01	I	I	I	I	1
6	ASFV Zabaykali WB-5314/2020	I	I	I	I	1
7	MK628478.1 ASFV/LT14/1490	II	I	I	I	2
8	CzechRepublic 2017/1	II	I	I	I	2
9	Moldova 2017/1	II	I	I	I	2
10	Belgium 2018/1	II	I	I	I	2
11	China/2018/AnhuiXCGQ	II	I	I	I	2
12	China Pig/HLJ/2018	II	I	I	I	2
13	China DB/LN/2018	II	I	I	I	2
14	Pol18_43035_O175	II	I	I	I	2
15	Pol19_55195-1_C2058/19	II	I	I	I	2
16	ASFV/Ulyanovsk 19/WB-5699	II	I	I	I	2
17	ASFV/Primorsky 19/WB-6723	II	I	I	I	2
18	Poland Pol16_29413_o23	I	I	II	II	3
19	Poland Pol16_20540_o10	II	I	II	II	4
20	Poland Pol17_04461_C210	II	I	II	II	4
21	Pol18_28784_O112	II	I	II	II	4
22	Poland Pol17_03029_C201	I	II	II	II	5
23	Pol17_55230_C728	II	II	II	II	6
24	Pol17_53450_C675	II	II	II	II	6
25	Pol18_43790_O180	II	II	II	II	6
27	ASFV/Kaliningrad 17/WB-13869	I	I	III	II	7
28	ASFV_Kaliningrad_18_WB-12523	I	I	III	II	7
29	ASFV_Kaliningrad_18_WB-12524	I	I	III	II	7
30	ASFV_Kaliningrad_18_WB-9735	I	I	III	II	7
31	ASFV_Kaliningrad_18_WB-9763	I	I	III	II	7
32	ASFV_Kaliningrad_18_WB-9766	I	1	III	II	7
33	ASFV_Kaliningrad_19_WB-10168	I	I	III	II	7
34	ASFV_Kaliningrad_18_WB-9734	I	I	III	II	7
35	ASFV_Kaliningrad_18_WB-12516	II	I	III	II	8

* Based on the 10 nt insertion in the IGR 173R/I329L, samples were divided in clusters IGR-1 and IGR-II (Figure 3).

** Based on the 14 nt insertion in ORF174L, samples were divided into clusters I and II (28).

*** Based on the two SNPs described in this study, sequences were divided into three clusters (I to III) (Figure 5).

**** Based on the SNP identified in this study, sequences were divided into two clusters (I to II) (Figure 4).

**Figure 6:**
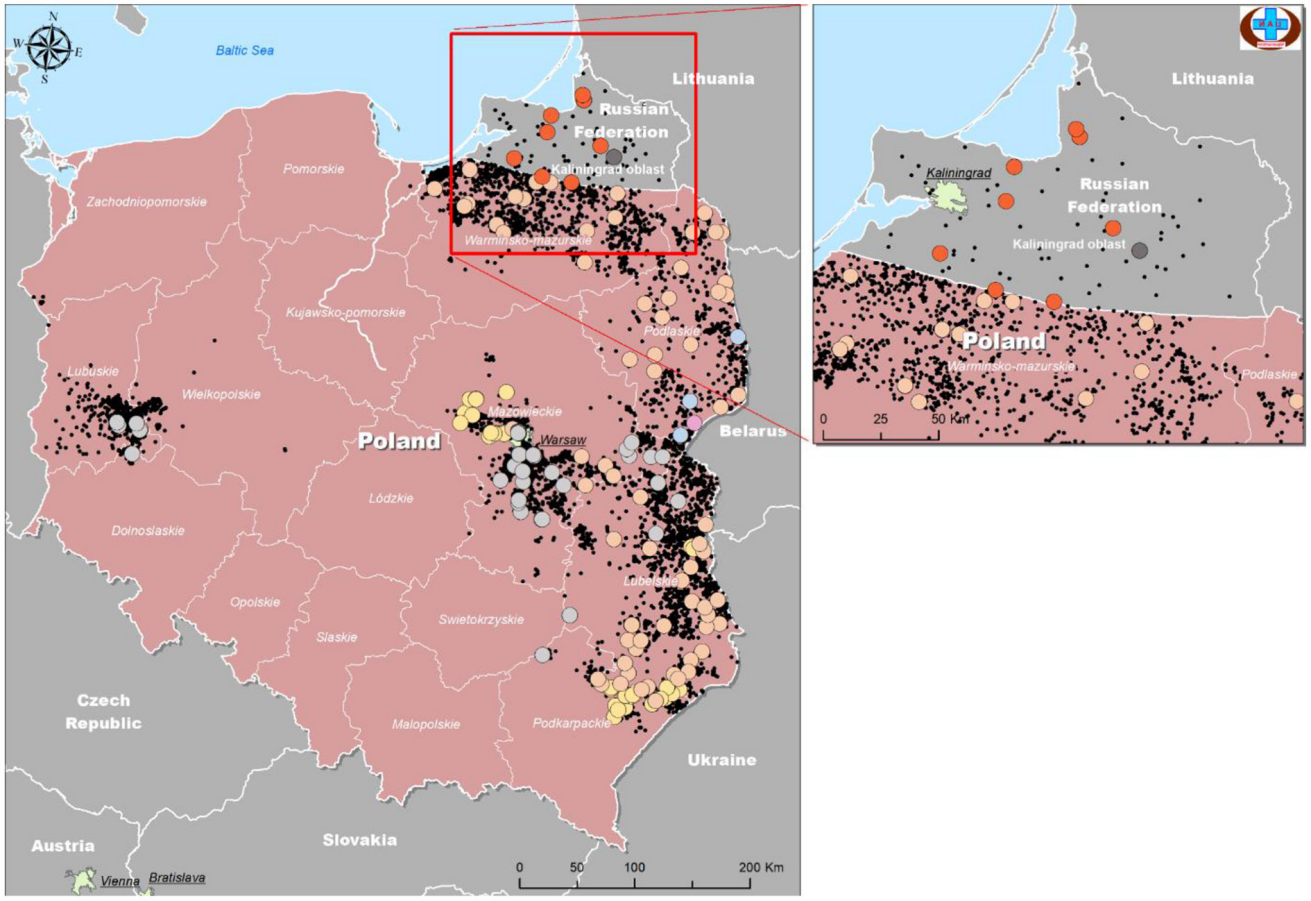
Geographical distribution of ASFV isolates from Kaliningrad and Poland based on analysis of genome markers IGR (I73R-I329L), O174L, K145R, and MGF 505-5R.

***** Each group number is denoted by a specific color similar to colors in Figure 6.

### ORF O147L

Since the sequences from Kaliningrad formed a close association with previously described isolates from Poland, it was important to investigate their relatedness. The importance of ORF O147L in dividing the samples isolated from Poland was previously described and was subsequently investigated in this study (28). None of the nine samples from Kaliningrad had the 14 nt insertion and thus clustered with the other isolates from the RF, and the majority of the samples from Poland, Europe, and Asia in cluster I (Table 4).

### Combined analysis of different genome markers

Encapsulating the informative SNPs of individual loci into groups and mapping the locations of each individual in a group can assist in identifying closely related isolates based on their geographical origins. The phylogenetic analysis previously described for the genome markers IGR (I73R-I329L), O174L, K145R, and MGF 505-5R was used to assign each of the ASFV genotype II isolates from the RF and Poland into eight different groups (Table 4).

Based on the aforementioned analysis, the results for SNPs or indels observed in the IGR between I73R and I329L, as well as the ORFs O174L, K145R, and MGF-505-5R, were summarized in a single table (Table 4). The nine isolates from Kaliningrad as well as sequences obtained from GenBank (Supplementary Table 2) were clustered into eight groups, each of which was assigned a unique color (Table 4). The nine isolates obtained from the Kaliningrad region clustered into two unique groups (7 and 8) (Table 4). This unique clustering of the Kaliningrad isolates was due to the SNP within K145R as well as the single isolate ASFV_Kaliningrad_18_WB-12516 clustering with IGR-II (Figures 3, 5). In contrast, isolates from Poland belonged to groups 2 to 6 (Table 4). Group 2 appears to be the dominant lineage and includes isolates from different geographical regions, including Poland, eastern and western Russia, China, Moldova, Latvia, Belgium, and Czechia.

The samples were mapped according to their location of origin as well as their assigned group based on Table 4. The colors used in Table 4 were repeated as a legend to identify each group, and information was used to plot isolates from Poland and Kaliningrad in order to estimate how isolates are distributed among regions and on the borders (Figure 6).

As demonstrated in Figure 6, the number of official outbreaks (black dots) in Poland was significantly higher than that in Kaliningrad. On the borders with Kaliningrad, the studied isolates did not match any of the groups circulating in the RF. Interestingly, each group has its own geographical distribution. For example, group four is mainly on the borders, while groups 2 and 3 approach the center of Poland. Group 7 was found in all regions of Kaliningrad, and group 8 only in one region about 30 km from the border with Poland.

## Discussion

In 2007, ASFV was first detected in Georgia, from where it massively and rapidly expanded into Russia (2007), Europe (2007), and Asia (2018) (29). Since the first report on ASFV in the RF in 2007, it spread throughout the country, reaching the region of Kaliningrad in 2017 (30). In addition to the continuous ASF pandemic in Eurasia (31), no reports of ASFV in Kaliningrad have been made since January 2020, but understanding the genomic profile of isolates circulating in Western and Eastern Europe will always be important for detecting and investigating any new outbreaks. It was suggested that the large-scale transmission of the virus between 2014 and 2017 throughout Poland and the Baltic countries (Lithuania, Latvia, Estonia) was facilitated by the high density of the wild boar population and the low level of biosecurity of backyard pig farms (32, 33). Wild boars were indeed shown to constitute a key factor in the persistence of ASFV in the wild and additional spillover into domestic pigs (34, 35). However, studies investigating wild boar populations in Western Europe suggest that human involvement played a more important role than dispersal by animals, since the latter stay within a few kilometers of their turf ranges, with little overlap among family groups (36).

In this study, we elucidated the whole coding genome sequences of nine ASFV isolates from wild boars detected in geographically separated locations of the Kaliningrad region at different time points during the period from 2017 to 2019. Additionally, these sequences were phylogenetically compared to ASFV from the EU, including Poland and the Baltic countries, as well as isolates from Russia and Asia (Figure 2). Interestingly, the new isolates proved highly similar to each other with >99.99% sequence identities over the whole genomes. Moreover, Kaliningrad isolates carry unique mutations in the K145R locus that have never been reported in any EU or Russian isolates and this unique mutation was confirmed by sequencing the K145R genome sequence by Sanger using specific primers (28). This could be indicative of novel genetic drift associated with region-specific viruses circulating in the wild boar population restricted to Kaliningrad. However, these isolates clustered as a sister lineage to the isolates obtained in Poland between 2016 and 2018. Although the range of European wild boars spans the Baltic countries, including Poland and Kaliningrad, it is important to note that the Kaliningrad-like isolates have not been reported in either Poland or Lithuania. This is likely attributable to stringent security measures restricting the migration of wild boars across administrative borders and also intrinsic behaviors of wild boars themselves (36). The sequences from Kaliningrad share the highest identity (>99.97%) to isolates from Poland, demonstrating a close geographical and evolutionary link with ASF throughout this region (Figure 2). This explains the observed clustering of Kaliningrad isolates with sequences from Poland based on the close proximity of the countries affected. The Kaliningrad/Poland cluster could be reliably distinguished by sequence analysis using SNPs in the three ORFs (MGF-505-5R, MGF-110-7L, and K145R) (Figures 4, 5). The unique mutations in K145R are indicative of the ongoing drift being exerted on the ASFV genomes; further sequencing of other isolates from the Kaliningrad region is required to further characterize and obtain a deeper understanding of the actual situation regarding ASF in the region. This difference between Polish and Russian isolates can be due to the time difference between the nine studied isolates and the 75 available Polish isolates in GenBank, or a lack of information about isolates from Poland, taking into consideration that only 16 isolates close to the Polish-Kaliningrad isolates were sequenced. Therefore, more sequencing should be performed in these areas to identify whether the two virus variants are co-circulating. Interestingly, strain ASFV/Kaliningrad/18WB-12516/Russia/2018 had a 10-nt (GAATATATAG) insertion within the IGR between I73R and I329L allocating it to group IGR-II, in contrast to the remaining eight sequences assigned to IGR-I (Figure 3). This was in contrast to four of the sequences from Poland belonging to IGR-I, whilst the other eight sequences clustered in IGR-II (Figure 3). This suggests a large population size consisting of multiple variants of both IGR-I and IGR-II circulating in the Baltic region. This is mirrored in the large number of SNPs acquired during the spread of genotype II ASFVs through Europe, Russia, and Asia (10).

This study reiterates the importance of whole-genome sequencing for elucidating viral epidemiology and evolution. Despite the close genetic relationship of the sequences from Poland with the nine isolates from Kaliningrad, the latter could be uniquely identified based on a single SNP in K145R. This is the first report of the distinguishing feature of K145R, following in-depth comparative analysis involving the whole-genome sequences.

## Data availability statement

The datasets presented in this study can be found in online repositories. The names of the repository/repositories and accession number(s) can be found in the article/Supplementary material.

## Ethics statement

The authors confirm that the ethical policies of the journal, as noted in the journal's author guidelines page, have been adhered to. Samples used in this study were those submitted to the Reference laboratory for African swine fever virus, FGBI Federal Centre for Animal Health (FGBI ARRIAH), for ASF diagnosis.

## Author contributions

Conceptualization: AM and ASc. Investigation: AM, AI, NZ, RC, ZD, and ASp. Data and analysis: AM, ASc, ASh, AI, ZD, FK, and ASp. Writing—original draft preparation: AM, ASc, and ASh. Writing—review and editing: AM, ASc, ASh, AI, FK, and ASp. All authors have read and agreed to the published version of the manuscript.

## Supplementary material

Supplementary Figure 1 **(A–C)** Maximum likelihood phylogenetic tree indicating the relationship of ASFV isolates based on their sequences of ORF MGF-360-10L **(A)**, ORF MGF-505-9R **(B)** and I267L **(C)**. The nine isolates obtained in Kaliningrad are indicated with black circles.

Supplementary Table 1 The calculated titer of individual ASFV isolates (*n* = 4) on cell culture, through three passages on PSC cells.

Supplementary Table 2 Data from GenBank of the ASFV genomes sequences used this study.
