# Supplementary figures and images for: Whole-genome sequencing of African swine fever virus from wild boars in the Kaliningrad region reveals unique and distinguishing genomic mutations
Source: Front Vet Sci. 2023 Jan 5;9:1019808. doi: 10.3389/fvets.2022.1019808 (PMC9849583; doi:10.3389/fvets.2022.1019808)

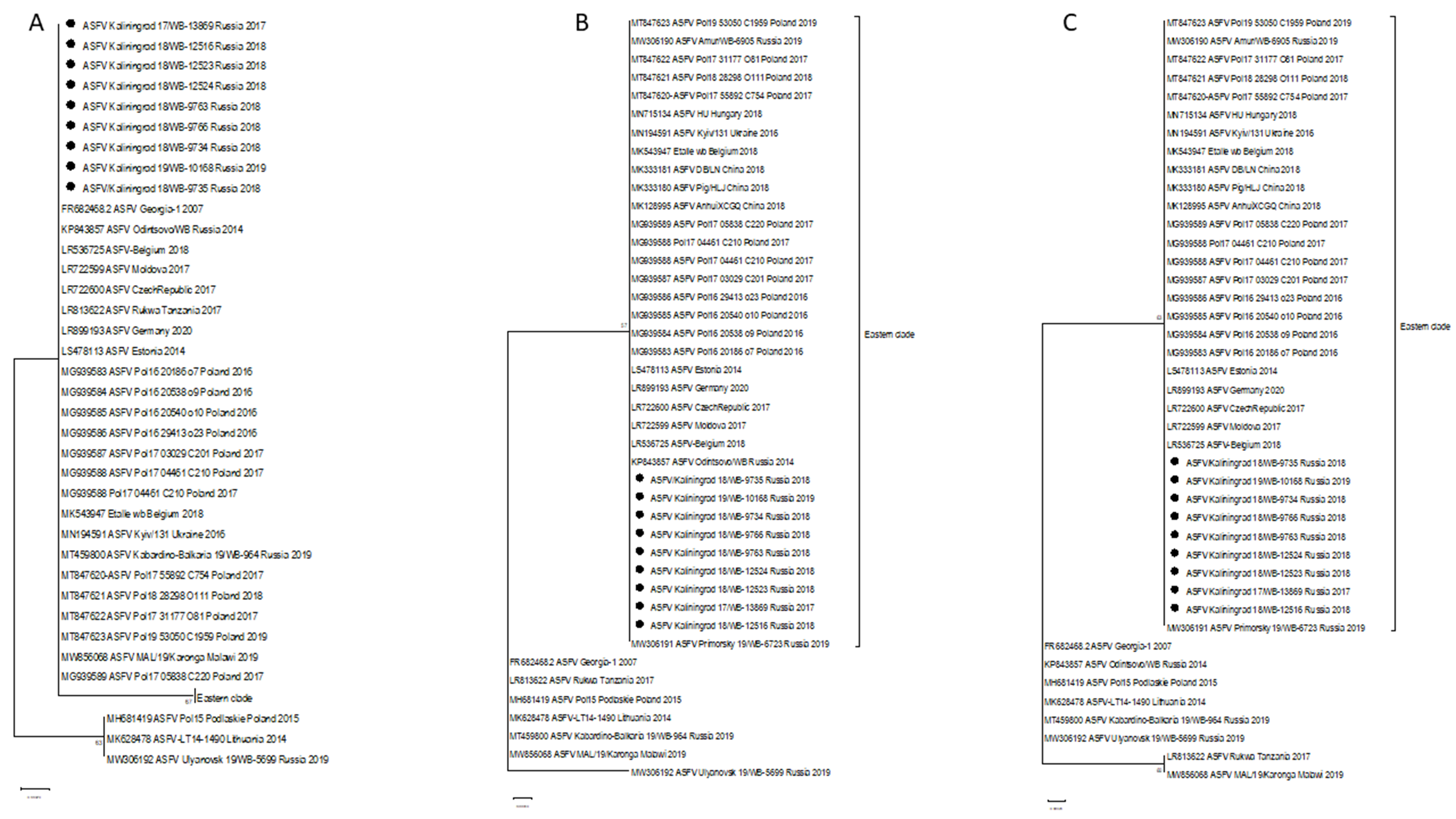

Supplement: Supplementary Figure 1 — (A–C) Maximum likelihood phylogenetic tree indicating the relationship of ASFV isolates based on their sequences of ORF MGF-360-10L (A), ORF MGF-505-9R (B) and I267L (C). The nine isolates obtained in Kaliningrad are indicated with black circles. [file Image_1.TIFF]
