# Supplementary material for: Whole-genome sequencing of African swine fever virus from wild boars in the Kaliningrad region reveals unique and distinguishing genomic mutations
Source: Front Vet Sci. 2023 Jan 5;9:1019808. doi: 10.3389/fvets.2022.1019808 (PMC9849583; doi:10.3389/fvets.2022.1019808)
Supplement: Supplementary Table 1 — The calculated titer of individual ASFV isolates (n = 4) on cell culture, through three passages on PSC cells. [file Table_1.DOCX]

Supplementary table 1. The calculated titer of individual ASFV isolates (n = 4) on cell culture, through three passages on PSC cells.

| Virus Isolate | **Virus Titer in Each Passage, lg HADU 50/cm3** ± **SD** | | |
| --- | --- | --- | --- |
|  | 1 | 2 | 3 |
| ASFV/Kaliningrad_17/WB-13869 | 4.22 ± 0.22 | 5.30 ± 0.10 | 5.90 ± 0.45 |
| ASFV/Kaliningrad_18/WB-9766 | 3.40 ± 0.50 | 4.85 ± 0.22 | 5.80 ± 0.50 |
| ASFV/Kaliningrad_18/WB-12523 | 5.20 ± 0.10 | 6.45 ± 0.30 | 7.10 ± 0.20 |
| ASFV/Kaliningrad_18/WB-9735 | 3.50 ± 0.25 | 4.20 ± 0.45 | 5.75 ± 0.10 |
| ASFV/Kaliningrad_18/WB-9734 | 2.80 ± 0.50 | 3.55 ± 0.35 | 5.10 ± 0.20 |
| ASFV/Kaliningrad_18/WB-9763 | 5.10 ± 0.20 | 6.50 ± 0.30 | 7.45 ± 0.25 |
| ASFV/Kaliningrad_18/WB-12524 | 4.90 ± 0.30 | 5.60 ± 0.10 | 6.90 ± 0.45 |
| ASFV/Kaliningrad_18/WB-12516 | 3.20 ± 0.70 | 4.90 ± 0.20 | 6.25 ± 0.30 |
| ASFV/Kaliningrad_19/WB-10168 | 4.00 ± 0.10 | 5.25 ± 0.32 | 7.10 ± 0.20 |
