# Supplementary material for: Whole-genome sequencing of African swine fever virus from wild boars in the Kaliningrad region reveals unique and distinguishing genomic mutations
Source: Front Vet Sci. 2023 Jan 5;9:1019808. doi: 10.3389/fvets.2022.1019808 (PMC9849583; doi:10.3389/fvets.2022.1019808)
Supplement: Supplementary Table 2 — Data from GenBank of the ASFV genomes sequences used this study. [file Table_2.DOCX]

Supplementary table 2. Data from GenBank of the ASFV genomes sequences used this study.

| Isolate name | Country of origin | Isolation date | GenBank accession number |
| --- | --- | --- | --- |
| Georgia/2007/1 | Georgia | 2007 | FR682468.2 |
| Rukwa/Tanzania/2017 | Tanzania | 2017 | LR813622 |
| MAL/19/Karonga/Malawi/2019 | Malawi | 2019 | MW856068 |
| T-L-1/Timor-Leste/2019 | Timor-Leste | 2019 | MW396979 |
| Estonia/2014 | Estonia | 2014 | LS478113 |
| LT14/1490/Lithuania/2014 | Lithuania | 2014 | MK628478 |
| Moldova/ 2017 | Moldova | 2017 | LR722599 |
| CzechRepublic/ 2017 | Czech Republic | 2017 | LR722600 |
| Etalle/wb/Belgium/2018 | Belgium | 2018 | MK543947 |
| wbBS01/China/2018 | PRC | 2018 | MK645909 |
| AnhuiXCGQ/China/2018 | PRC | 2018 | MK128995 |
| Pig/HLJ/China/2018 | PRC | 2018 | MK333180 |
| DB/LN/China/2018 | PRC | 2018 | MK333181 |
| Wuhan-1/China/2019 | PRC | 2019 | MN393476 |
| Wuhan-2/China/2019 | PRC | 2019 | MN393477 |
| pig/CAS19/China/2019 | PRC | 2019 | MN172368 |
| CN/2019/InnerMongolia/AES01/China/2019 | PRC | 2019 | MK940252 |
| CADC/HN09/China/2019 | PRC | 2019 | MZ614662 |
| HuB20/China/2020 | PRC | 2020 | MW521382 |
| Pig-HRB1/China/2020 | PRC | 2020 | MW656282 |
| Germany/2020 | Germany | 2020 | LR899193 |
| Pol15/Podlaskie/Poland//2015 | Poland | 2015 | MH681419 |
| Pol16/20186/o7/Poland/2016 | Poland | 2016 | MG939583 |
| Pol16/20538/o9/Poland/2016 | Poland | 2016 | MG939584 |
| Pol16/20540/o10/Poland/2016 | Poland | 2016 | MG939585 |
| Pol16/29413/o23/Poland/2016 | Poland | 2016 | MG939586 |
| Pol17/03029/C201/Poland/2017 | Poland | 2017 | MG939587 |
| Pol17/04461/C210/Poland/2017 | Poland | 2017 | MG939588 |
| Pol17/05838/C220/Poland/2017 | Poland | 2017 | MG939589 |
| Pol17/55892/C754/Poland/2017 | Poland | 2017 | MT847620 |
| Pol18/28298/O111/Poland/2018 | Poland | 2018 | MT847621 |
| Pol17/31177/O81/Poland/2017 | Poland | 2017 | MT847622 |
| Pol19/53050/C1959/Poland/2019 | Poland | 2019 | MT847623 |
| Odintsovo/WB/Russia/2014 | Russia | 2014 | KP843857 |
| Amur/WB-6905/Russia/2019 | Russia | 2019 | MW306190 |
| Primorsky/19/WB/6723/Russia/2019 | Russia | 2019 | MW306191 |
| Ulyanovsk/19/WB/5699/Russia/2019 | Russia | 2019 | MW306192 |
| Kabardino-Balkaria/19/WB/964/Russia/2019 | Russia | 2019 | MT459800 |
| VN/QP/Vietnam/2019 | Vietnam | 2019 | MT882025 |
| Hanoi/Vietnam/2019 | Vietnam | 2019 | MT166692 |
| NgheAn/Vietnam/2019 | Vietnam | 2019 | MT180393 |
